# Customized virtual reality naturalistic scenarios promoting engagement and relaxation in patients with cognitive impairment: a proof-of-concept mixed-methods study

**DOI:** 10.1038/s41598-023-47876-1

**Published:** 2023-11-22

**Authors:** Susanna Pardini, Silvia Gabrielli, Lorenzo Gios, Marco Dianti, Oscar Mayora-Ibarra, Lora Appel, Silvia Olivetto, Alina Torres, Patty Rigatti, Emanuela Trentini, Lucia Leonardelli, Michela Bernardi, Marzia Lucianer, Stefano Forti, Caterina Novara

**Affiliations:** 1 Department of General Psychology, University of Padova, Padua, Italy; 2 Digital Health Research Unit, Centre for Health and Wellbeing, Fondazione Bruno Kessler, Trento, Italy; 3 Human Inspired Technology Research Centre (HIT), University of Padova, Padua, Italy; 4 Competence Center for Digital Health, TrentinoSalute4.0, Trento, Italy; 5 OpenLab, University Health Network, Toronto, ON Canada; 6 Faculty of Health, School of Health Policy and Management, York University, Toronto, ON Canada; 7 Michael Garron Hospital, Toronto, ON Canada; 8 Faculty of Science, University of Toronto, Toronto, ON Canada; 9 Azienda Pubblica di Servizi alla Persona (APSP) “Margherita Grazioli”, Trento, Italy

**Keywords:** Human behaviour, Quality of life, Therapeutics

## Abstract

Being immersed in a natural context has a beneficial and pervasive impact on well-being. Virtual Reality (VR) is a technology that can help expose people to naturalistic scenarios virtually, overcoming obstacles that prevent them from visiting real natural environments. VR could also increase engagement and relaxation in older adults with and without cognitive impairment. The main aim of this study is to investigate the feasibility of a customized naturalistic VR scenario by assessing motion-sickness effects, engagement, pleasantness, and emotions felt. Twenty-three individuals with a diagnosis of cognitive impairment living in a long-term care home participated in our study. At the end of the entire VR experimental procedure with older adults, five health staff operators took part in a dedicated assessment phase focused on evaluating the VR procedure's usability from their individual perspectives. The tools administered were based on self-reported and observational tools used to obtain information from users and health care staff professionals. Feasibility and acceptance proved to be satisfactory, considering that the VR experience was well-tolerated and no adverse side effects were reported. One of the major advantages emerged was the opportunity to deploy customized environments that users are not able to experience in a real context.

*Trial Registration*: National Institute of Health (NIH) U.S. National Library of Medicine, ClinicalTrials.gov NCT05863065 (17/05/2023).

## Introduction

There is growing evidence of the positive impact of being immersed in natural environments on physical and mental health^1–3^. Natural contexts increase the perception of positive emotions, reduce fear, anxiety, anger, and sadness, and play an essential role in regulating heart rate, blood pressure, muscle tension, and brain electrical activity^4^.

In healthcare facilities, allowing older adults to have recreational and pleasant experiences in natural environments, especially if they have a cognitive and physical impairment, although challenging, can be beneficial^5^. Participating in organized outdoor activities may be limited by reduced motor ability and/or cognitive impairment of the patients. In addition, outdoor spaces could not always be available. Overall, evidence shows that even if unable to engage directly in a natural environment, visible exposure to nature scenes can be beneficial, reducing hospital length-of-stay and drug consumption^5–8^. Based on these findings, it is desirable to identify alternative strategies for providing older adults with exposure to natural scenes while accounting for barriers to mobility, autonomy, and safety concerns.

A potential innovative method helpful in overcoming these obstacles could be the deployment of Virtual Reality (VR). VR technologies have been demonstrated to increase engagement across several populations^9^. VR systems consist of technologies that provide the user with multiple sensory inputs through, for example, visual and auditory displays and can have varying degrees of immersion. Immersive VR consist in a three-dimensional environment that is simulated through a computer's memory, graphics, and processes. In the clinical psychological field, it is often used to create simulated environments for activities such as flying a plane or exploring space, which are expensive or dangerous to experience directly. Hardware and software tools, such as gloves and head monitors with real-time feedback, are frequently utilized to immerse and train humans in these virtual scenarios. Indeed, VR could take variant forms, ranging from desktop computer rendering of a highly interactive virtual world to a fully immersive multisensory environment in laboratories^10,11^.

Immersive visual information is generally conveyed through displays, where the interface allows exposure to content that can be explored in 360-degrees, as with head-mounted displays (HMDs). Auditory information can be provided via headphones some of which are embedded directly into the HMD. VR-based interventions have been successfully deployed for addressing a myriad of clinical conditions such as specific phobia, social anxiety, panic disorder, post-traumatic stress disorder, and depressive disorders^12–16^.

Evidence suggests that immersive virtual scenarios can improve the quality of life in older adults' healthcare facilities, highlighting a positive impact on behavioral and psychological symptoms. VR was also found to overcome some barriers that can hinder participation in recreational activities, such as the difficulty of moving in the environment^17–19^. A study by Briemelow et al.^20^ demonstrated how immersive scenarios can positively impact users with heterogeneous cognitive impairment levels living in a long-term care home with minimal negative effects.

Patients with cognitive decline may benefit from improving cognitive, emotional, and physical fitness functions if exposed to multisensory VR contexts characterized by quasi-naturalistic and realistic stimuli^21–24^. To our knowledge, few studies have investigated the feasibility and effectiveness of using immersive virtual environments for managing anxiety and promoting a state of relaxation in populations of elderly individuals with impaired physical and cognitive functions^18,25,26^. Moreover, there is a lack of research on the customization of virtual scenarios for therapeutic purposes.

Some published research has highlighted how using VR HMD promotes the management of depressive and anxious symptoms in older people^27,28^. Appel et al.^18^ investigated the relationship between relaxing, positive environments and both positive and negative emotions based on a sample of older adults with varying (mild to severe) cognitive and physical impairment levels. The participants were exposed through an HMD for an average variable period of 8 min to a relaxing environment in which realistic natural scenarios were depicted. All 66 participants completed the research without experiencing or reporting adverse side effects related to using the HMD. Qualitatively, positive feedback is associated with the experience and the perception of a higher state of relaxation. Additionally, there has been an increase in the intensity and frequency of positive emotions experienced (e.g., feelings of relaxation and happiness) and a reduction in the levels of some negative emotions (e.g., sadness and anxiety)^18^. The authors concluded that exposure of people with cognitive and physical impairments to realistic and natural immersive scenarios in virtual reality through an HMD is safe and feasible. These findings encourage future studies to investigate the role of virtual reality scenarios’ customization.

Additional research by Moyle et al.^29^ exposed individuals with dementia living in care facilities to a visual and auditory interactive virtual experience with the aim of improving the quality of life by obtaining information about users’ moods, apathy, and engagement. The exposure consisted in observing a screen, without using an HMD, in which a natural scenario was represented. During the exposure, participants were able to select seasons (spring and autumn) and different types of virtual objects through hand and arm movements. Residents, family members, and staff have received positive feedback globally. Participants experienced higher levels of engagement, pleasure, activation, and fear during the exposure to the virtual forest than the control sample. Of note, the authors stated that differences in users’ preferences should be considered as a main factor limiting the versatility of the VR Forest.

Research on the effect of customizing VR-scenarios has grown in recent years^30,31^, uncovering benefits such as an increased sense of presence and engagement in the virtual environment. A relaxing and customizable VR environment implies an a priori identification of conditions that can be pleasant and relaxing based on the user's needs, allowing the management of any interfering environmental factors that might also be present in the real context^30,32,33^. This person-centered approach is especially valuable for those living with cognitive impairment as it also provides a sense of familiarity and security within the VR context^34^. but additional investigations are still needed to obtain more consistent data on its feasibility and effectiveness in this population.

Considering the promising outcomes of recent studies (e.g.,^18,19,29^) and the recognition that more depth (through rigorous research), and more breadth (encompassing new elements of design, e.g., customization) are needed for VR-interventions to be adopted by mainstream healthcare, we designed a proof-of-concept study, with the following goals:

- The primary objective is to evaluate the impact of VR on self-reported and observational levels of motion-sickness, engagement, and pleasantness in older adults living with cognitive impairment and residing in long term care.
- Based on our knowledge, distinct from most studies in this field, the current study intended to provide customization of visual and audio stimuli over and above offering a selected number of outdoor settings. For this reason, the secondary aim is to investigate if customized, relaxing virtual environments can be acceptable to the target group, as well as positively impact feelings and state anxiety, at least in the framework of a proof-of-concept study. Indeed, this study is part of a continuum of research on the requirements and effects of VR customization for different target populations. The study leveraged on the positive results of VR customization collected in a previous study we conducted^29^ involving non clinical population and it intended to further explore the feasibility of different customization options.
- A third goal is to investigate the usability of the VR apparatus from the perspective of health staff working in long term care to better understand the acceptance and potential adoption of the proposed solution in these clinical settings.

## Methods

The current proof-of-concept and feasibility study is a one-session single-centre trial conducted at the *Azienda Pubblica di Servizi alla Persona (APSP) “Margherita Grazioli*”, a long-term care home in Trento (Italy) in collaboration with the Department of General Psychology—University of Padova (Italy) and the Centre for Health and Wellbeing-Fondazione Bruno Kessler (Italy). The proposed study protocol was approved by the Ethical Committee for Clinical Experimentation—*Azienda Provinciale per i Servizi Sanitari*—*Provincia Autonoma di Trento* (Italy), (Nr: A827, 10/2022, Rep. Int. 20,298 11/22/2022). The research complies with the relevant ethical regulations of the Declaration of Helsinki.

The study design is based on a mixed-methods approach inspired by the Obesity-Related Behavioral Intervention Trials (ORBIT) framework^35^ for the design (Phase Ib) of digital interventions and their preliminary testing (Phase IIa). At the current stage, a sample is usually selected from accessible individuals to understand if the intervention deserves an increased depth of analysis, improvement, and future testing.

## Participants

**Figure 1:**
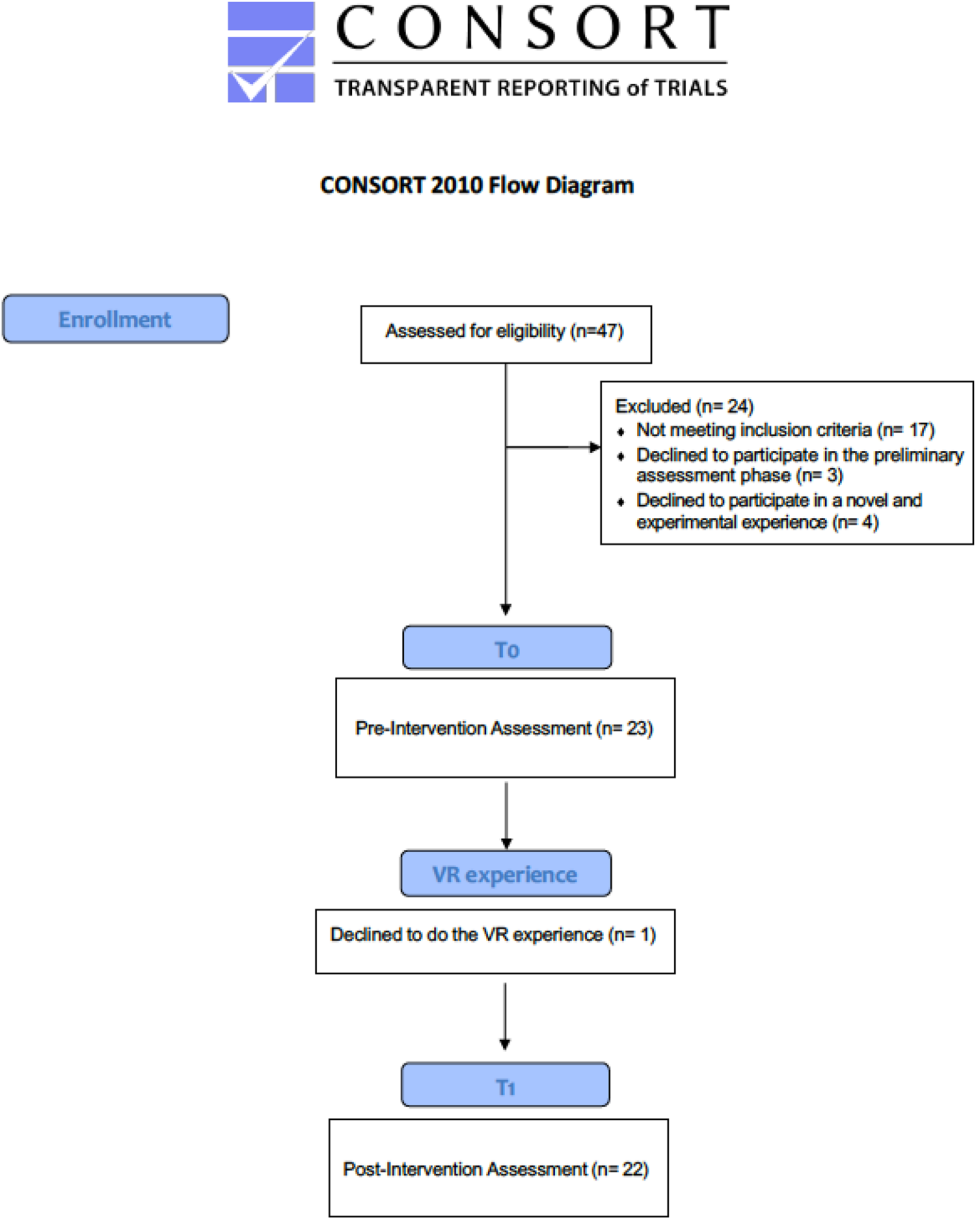
CONSORT 2010 flow diagram—adapted for the current study Source .^36^

An APSP healthcare professional (*Educatrice socio-assistenziale*) identified individuals eligible for the study based on an ad hoc schedule and obtained informed consent. Written informed consent was collected from all participants or their Substitute Decision Makers (SDMs). In addition, authorization by the primary care physician was also requested. Patients over 18, Italian mother tongue, and with mild, moderate, or severe cognitive impairment were eligible for participation. Exclusion criteria were: (1) palliative care; (2) diagnosis of psychosis; (3) severe neurological damage; (4) a positive diagnosis of epilepsy or having first-degree relatives diagnosed with epilepsy; (5) cardiac pacemaker or other metal devices; (6) infectious or gastrointestinal disorders; (7) the presence of open wounds at the level of the face; (8) motor or visual dysfunctions and neuromuscular pain that prevent the use of Oculus. Participants did not receive remuneration upon completion of the study. Overall, 47 users were considered for participation in the study. Of these, 24/47 did not participate for different reasons: (1) 17/47 did not meet all the criteria of inclusion/exclusion; (2) 3/47 did not agree to make the preliminary assessment phase; (3) 4/47 did not want to participate because they did not want to try a new experience that needed to be further studied to better understand the effects. The final sample was composed of 23/47 users, one of whom was not exposed to Oculus for fear of wearing the HMD (see Fig. 1).

## Intervention

### Hardware and software equipment

A commercially available VR HMD (Oculus Quest 2, Meta Quest)^37^, Alienware m15 Ryzen Edition R5″ workstation, and a link cable were used. The virtual scenarios were developed in the Unity framework by using the C# programming language (source of assets: https://freesound.org/; Unity; Polyhaven; HDRIhaven). The code was versioned via GitLab. The protocol for the virtual environment design in the current study has been outlined by Pardini et al.^31^ The individual sessions were video-recorded to allow for triangulation, including nonverbal behavior. Most of the experimental sessions were conducted in the dedicated music-therapy room at the APSP. All of the sessions were administered and evaluated by the same assessor. Health workers accompanied and passively assisted the procedure as observers to get information on the feasibility and usefulness of the procedure and to inform subsequent studies and system design with their requirements. Observations from health professionals were collected through the SUS, and the MARS questionnaires and by a dedicated Focus Group session.

To manage problems related to physical movements and fear of going into an unfamiliar context, two individual sessions were administered in a more commonplace setting, and for one participant, the procedure was administered in their private room. Each session lasted approximately 50 min. For safety reasons, VR was always administered when participants were seated safely in a chair or wheelchair. To allow individuals to explore the virtual context, the experimenter and the health care professional who assisted the patient during the procedure, helped the participants to rotate their heads and move their chair or wheelchair.

**Figure 2:**
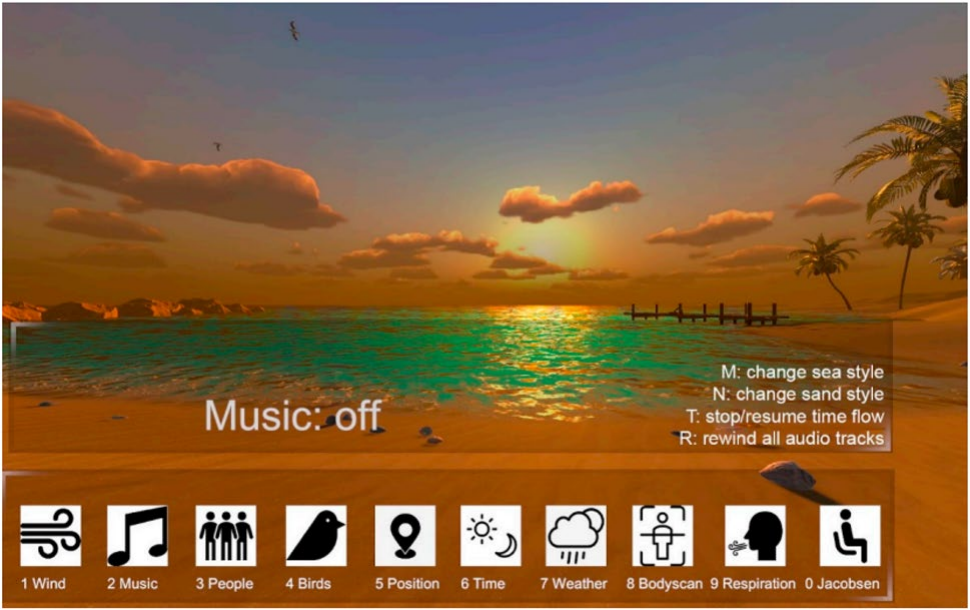
The PC’s interface with icons related to the different customizable variables (e.g., music, wind, weather conditions, time of day, and presence of people)^32^. The image was taken by authors of the current study.

**Figure 3:**
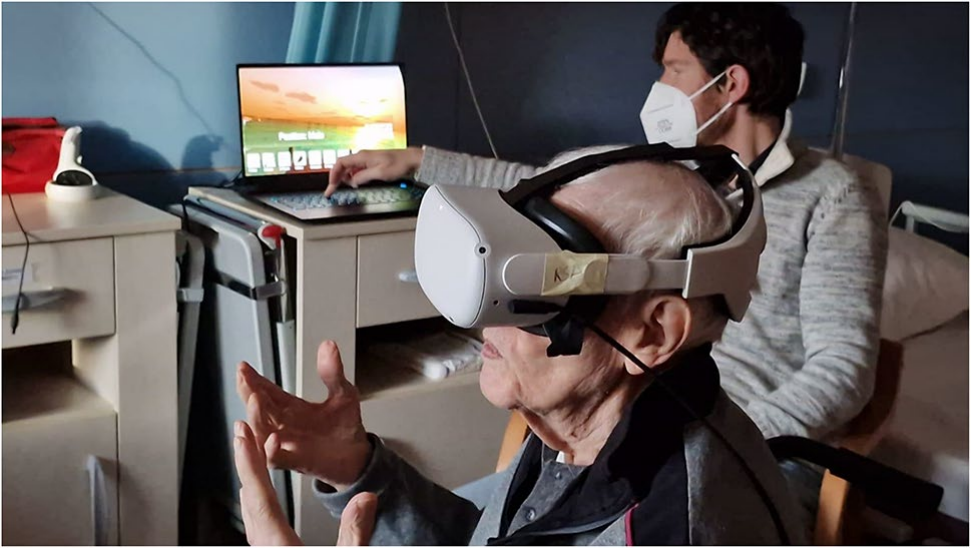
A participant during the experimental phase with Virtual Reality. Written, informed consent was obtained from the individual or their SDMs to publish these images. Fondazione Bruno Kessler and TrentinoSalute4.0 have the copyright, and permission must be obtained for use in other sources. The image was taken by authors of the current study.

A brief baseline assessment (T0) investigating the ability to move head and body, the use of visual and auditory aids, and the general emotional state were conducted based on an ad hoc measure and a modified version of the State-Trait Anxiety Inventory- Y1 (STAI-Y1) inspired to the version developed by Appel et al.^18,19^ At least 2 min of VR exposure were conducted before the trial to familiarize patients with the virtual context. With technical operator support, participants could select and customize all the audio and video stimuli (see Fig. 2). A specific range of customization options was chosen as appropriate for the target group of the study, also relying on results of previous studies^27^. Participants had the possibility to visualise the different options and to ask the researchers to modify specific characteristics of the environment. The technical operator and the experimenter could customize virtual scenarios based on a PC’s dashboard interface connected to Oculus Quest 2 via a cable. Specifically, the interface presented a series of icons related to the different customizable variables (such as music, wind, weather conditions, time of day, and presence of people) (Fig. 2). The connection between the PC and the viewer allowed the operator to see what the participant was observing during the experience. Participants had the possibility to visualise the different options and to ask the researchers to modify specific characteristics of the environment. Then, the participant was told they could remove the HMD when desired. Throughout the VR session, the experimenter took notes of the participants’ verbal and non-verbal communication based on the Observed Emotion Rating Scale (OERS), and an Ad Hoc Observation Form. Post the intervention phase (T2), the following measures were collected: emotional state, motion-sickness symptoms, usability, engagement, and pleasantness experienced during the activity. Considering the difficulties participants with cognitive impairment could have in peculiar situations, and moments of the day (e.g., negative symptoms relating to the sundowning syndrome), each VR session was arranged based on the participants’ and health staff’s needs and preferences. Figure 3 present a picture of a user during their VR experience.

At the end of the entire experimental phase with all participants, an assessment based on self-report questionnaires and a focus group was conducted with the health staff professionals that observed and assisted the older adult participants during the VR experience.

### Sample size estimation

Even if based on the experimental design, the estimation of sample size does not require, at this level of investigation, to have more rigorous information for interpreting in a more conservative way quantitative data, sample size calculations were performed. This permits to estimate the sample size needed to detect a significant difference based on the administration of the STAI: for this reason, an a priori power analysis has been executed determining an expected medium standardized effect size (effect size = 0.65), with a power of 80%. Considering a non-normal distribution of the STAI, sample size was calculated with G-Power for non-parametric tests (Kruskal–Wallis Test, Wilcoxon test). Results suggest that a sample of at least 22 patients have to be enrolled. Sample size and Power for non-parametric tests (Kruskal–Wallis Test, Wilcoxon test) were calculated with G-Power. Results suggest that, with a non-normal distribution, 22 patients have to be enrolled. If parametric statistics are conducted (assuming the normal distribution), the sample should instead consist of *n* = 21 users with Bonferroni correction (alpha = 0.05), an effect size equal to 0.65, and a power of 80%. Considering the current sample was not normally distributed based on the STAI, a sample of at least 22 patients is enrolled.

### Outcome measures

*Data collection.* Measures are administered in the form of interviews or as observation administered before, during, and after the VR experience. Questionnaires were administered verbally, and participants were asked to respond when they could. Information about the participant's current mental and physical conditions were extrapolated from their medical record.

A **Socio-demographic questionnaire** was administered by the health care professional to obtain information about gender, age, marital status, past occupation, degree level, proneness to motion sickness symptoms, hearing and vision impairments, mobility limitations or use of physical aids, previous experience with VR, and years of stay at the APSP facility. Moreover, the health care professional obtained information about the cognitive impairment level and psychological symptoms from the medical record (e.g., Mini-Mental State Examination Score-MMSE^38,39^, and the U.C.L.A. Neuropsychiatric Inventory-NPI^40^). The inclusion and exclusion criteria evaluation was performed with the demographic schedule.

The modified version of the **State-Trait Anxiety Inventory-Y1 (STAI-Y1)**, inspired by Appel et al.^18,19^ was administered in this study to obtain information on the state-anxiety level and other types of emotions individuals experienced before and after the VR session. The current study specifically investigates relaxation, worry/anxiety, sadness, tightness, annoyance, and anger. The scale was administered as an interview based on a 5-point Likert scale from 1 (not at all) to 5 (a lot). For the current study, Cronbach’s α displayed an adequate consistency for the total score recorded at T0 (Cronbach’s *α* = 0.75) and at T1 (Cronbach’s *α* = 0.71).

The experimenter used the Observed Emotion Rating Scale (OERS) during the VR session. It was adapted from the original version of Lawton et al.^41^ and used as an observation tool to assess the presence and frequency of negative emotions (fear, anxiety, anger, and sadness) and positive (pleasure) feelings experienced during the session based on a scale from 1 to 5 (1: “undetected emotion”; 2: “emotion observed for less than 16 s”; 3: “emotion observed for 16–59 s”; 4: “emotion observed for 1–5 min”; 5: “emotion observed for more than 5 min”).

During the VR session, the Ad Hoc Observation Form was also completed to record the type and frequency of vocalizations, phrases, facial expressions, body movements, reminiscences, and pleasant memories elicited during the VR exposure.

The Virtual Reality Symptom Questionnaire (VRSQ^42,43^) assesses the general and eye-related physical symptoms of exposure to a virtual reality environment. The score assigned to each item ranges from 0 to 6, with a maximum total score of 84 (48 for general symptoms and 36 for eye symptoms). Higher scores represent worse symptoms, with 0 corresponding to no adverse effects, and 84 to serious adverse effects.

The VR experience tolerability was primarily estimated based on the frequency of time spent in the VR context, on positive and negative emotion expressed (such as boredom, joy) and the weight of the HMD. Finally, the usability, engagement, pleasantness, and satisfaction experienced in using the VR apparatus were inspired by a measure developed and described by Appel et al.^18,19^ composed of both self-reported questions and other queries that the experimenter answered by observing the participant’s behavior during the experience and characterized by a series of items based on a 5 points Likert scale (Questions about Level of interest, awareness, engagement, and enjoyment observed: 1 = “very much”, 5 = “not at all”; questions on other information in relation to the VR experience: 1 = “strongly disagree”, 5 = “strongly agree”), and six open-ended questions focalized in obtaining additional information, where possible, about: 1) what participants liked best and least; 2) what participants would like to see; 3) if participants would like to repeat the experience; 4) if participants would recommend the experience to a friend.

To obtain information from health professionals working in the APSP about usability and the perceived quality rating of the VR set-up deployed, the Adapting-Mobile App Rating Scale (A-MARS)-Subjective Quality Scale^44^, and the System Usability Scale (SUS)^45–47^ were filled in by operators who participated during the VR sessions. Questionnaires’ administration was performed after the administration of the experimental procedure. At the end of the experimental phase, a focus group was conducted with the health professionals that assisted users during the VR experience. Issues discussed during the focus group were the strengths and weaknesses associated with using virtual reality, the future perspectives, and the risks associated with using virtual reality with users affected by cognitive impairment.

### Data analysis

Continuous data are presented as mean ± standard deviation. Categorical data are presented as numbers with percentages. The statements made by participants during the VR experience were analyzed by SP and SO using thematic analysis and reported as frequencies. Data were analyzed thematically following an inductive, data-driven approach^48^. Data codes were generated systematically, collated into themes, and applied to the data set to generate frequencies. For a comparison of non-normally distributed continuous variables before and after the VR intervention, the Wilcoxon signed-rank test is used for each intervention separately. A two-sided *p*-value < 0.05 is considered significant. Statistical analyses were undertaken using SPSS® version 29^49^. Moreover, qualitative responses were transcribed before, during, and after the VR exposure. In order to analyze data obtained by the audio-recorded focus group, the micro interlocutor analysis method has been applied^50^. The present method is helpful in obtaining information on participants’ attitudes, points of view on the use of VR, and permits to have quantitative data on participant grouping. Data were analyzed based on descriptive statistics.

## Results

### Demographic analysis

Twenty-three older adults were recruited from the APSP “Margherita Grazioli”. Demographics and clinical features of the study sample are described in Tables 1 and 2.

**Table 1:** Demographic features of the entire sample.

Demographic features	Sample group
	N** = 23**
Gender	N (%)
Women	19 (82.6%)
Age	Mean (SD)
	86.6 (5.12)
Marital status	N (%)
Single	1 (4.3%)
Married	5 (21.7%)
Divorced	2 (8.4%)
Widowed	15 (65.2%)
Education	N (%)
Primary school	13 (56.5%)
Middle school	3 (13%)
High school	3 (13%)
Employability training certificate	4 (17.4%)
Past employment	N (%)
Housekeeper	11 (47.8%)
Worker	7 (30.4%)
Clerical worker	3 (13%)
Teacher	2 (8.6%)
Aids	N (%)
Wearing glasses	12 (52.2%)
Hearing problems	6 (26.1%)
Wheelchair user	11 (47.8%)
Walking aid	6 (26.1%)
Multifunctional highchair	1 (4.3%)
Walking stick	1 (4.3%)
No aids	1 (4.3%)
Dizziness and head-turning over moving locomotion	N (%)
Yes	None
Sickness experienced on moving locomotion	N (%)
Yes	0
Sometimes	6 (26.1%)
No	17 (73.9%)
Feel involved in watching television	N (%)
Yes	13 (56.5%)
A little	5 (21.7%)
No	5 (21.7%)
Aware of what VR is	N (%)
Yes	2 (8.7%)
No	21 (91.3%)
Past experiences with VR	None
Yes	
Favourite actual pleasant activities	N (%)
Listen to music	2 (8.7%)
Reading	2 (8.7%)
Walking	6 (26.1%)
Dancing	1 (4.3%)
Talking with friends	2 (8.7%)
Painting	1 (4.3%)
Playing card games	1 (4.3%)
Doing crossword puzzles	1 (4.3%)
Watching TV	6 (26.1%)
Outdoor activities	1 (4.3%)
Activities to cope with anxiety (in the past)	N (%)
Painting	1 (4.3%)
Outdoor activities	14 (60.9%)
Knitting	2 (8.7%)
Reading	1 (4.3%)
Listen to music	1 (4.3%)
Spending time with family and friends	2 (8.7%)
Singing	1 (4.3%)
To pray	1 (4.3%)

**Table 2:** Clinical features of the entire sample (*N* = 23).

Mini-mental state examination (MMSE)		
Cognitive impairment levels (range)		N (%)
Mild (18–26)		
(n, %)		11 (47.8%)
Moderate (10–17)		
(n, %)		9 (39.2%)
Severe (< 10)		
(n, %)		3 (13%)
Neuropsychiatric inventory (NPI)		
Clinical features	N (%)	(Frequency x Severity)
		Mean (SD)
Delusions	0	0
Hallucinations	0	0
Agitation/Aggression	2 (8.7%)	0.43 (1.47)
Depression/Dysphoria	7 (30.4%)	1.52 (3.12)
Anxiety	9 (39.2%)	2.57 (4.09)
Elation/Euphoria	0	0
Apathy/Indifference	4 (17.4%)	1.22 (3.45)
Disinhibition	3 (13%)	0.91 (2.94)
Irritability/Lability	7 (30.4%)	1.30 (2.98)
Aberrant motor behavior	5 (21.7%)	1.61 (3.81)
Sleep and Nighttime Behavior Disorders	5 (21.7%)	1.30 (3.11)
Appetite and Eating Disorders	2 (8.7%)	0.87 (2.94)

It is important to specify that twenty individuals (87%) could move their heads without difficulty, and only 5 (34.8%) were able to move autonomously in the environment, but with supervision. Eleven participants were in a wheelchair during the VR experience (47.8%), and twelve (52.2%) used the chair provided by researchers.

In order to control the potential relationship between the presence of neuropsychiatric symptoms and the cognitive impairment level, Kendall’s tau rank and Spearman’s rho correlations between the MMSE and the NPI total score were executed considering the entire sample. No relationship between neuropsychiatric symptoms and the cognitive impairment level emerged (Kendall’s tau rank = − 0.23; *p*-value > 0.05; Spearman’s rho = − 0.29; *p*-value > 0.05).

### 1^ objective: Investigation of tolerability, motion-sickness effects, engagement, and pleasantness associated with the VR experience

VR tolerability was operationalized as the frequency of time spent in the VR context that is also dependent on other constructs related to the emotions felt during the experience and the wearability of the HMD. On average, participants spent 9.91 min exposed to the VR scenario (SD = 4.78; minimum = 1 min; maximum = 17 min). Fifteen users (68.19%) spent at least 9 min of time in the VR context (SD = 4.78; minimum = 1 min; maximum = 17 min). None of the users referred to experiencing a general condition of discomfort, drowsiness, headache, sweating, claustrophobia or disorientation, and nausea during the VR experience. Three participants 3/22 (13.1%) felt a slight fatigue state, 1/22 (4.3%) experienced boredom, 10/22 (43.5%) reported that their eyes were slightly tired and irritated during the VR exposure, 2/22 (8.7%) experienced a slightly blurred vision, and 3/22 (13%) reported having had slight difficulty focusing. Ten (43.5%) participants experienced discomfort due to wearing the HMD (two participants said: “*these special glasses are heavy*”), and one participant (%) completed only the T0 assessment but decided not to try the VR since he was worried about wearing the HMD.

Table 3 summarizes the responses assessed on a 5-point Likert scale, from 1 (very much) to 5 (not at all), related to the interest, awareness, engagement, and enjoyment shown during the VR experience highlighting how the VR activity, on average, was appreciated by participants.

**Table 3:** Level of interest, awareness, engagement, and enjoyment observed.

Interest and awareness	Mean (SD)
General interest in the visual and auditory stimuli of the VR scenario	1.85 (0.75)
Awareness during the experience (based on posture and facial expression)	1.85 (0.67)
Engagement	Mean (SD)
Engagement (based on verbal feedback)	1.95 (0.83)
Reminiscences	2.20 (1.20)
Enjoyment	Mean (SD)
Enjoyment (based on posture and facial expression)	1.20 (0.41)

Some of the verbal feedback (direct quotes) about engagement in VR were: “*beautiful”, “I would like to add other flowers”, “I am feeling good here”, and “It is marvelous”.* Two users said, *“the experience was good, but it should be useful for younger people”,* and *“not bad (…), but I prefer to do other things ”*.

During and after the experience, 11/22 participants (50%) reported that the VR scenario triggered memories by saying, for example: *“When I was younger, I went to Sicily with my babies”, “It is different (but) I am thinking about the picnic at the mountain with friends”.* Moreover, post-intervention, a participant said that the experience allowed her not to think about negative things for a while. In general, 16/22 users (72.7%) expressed their desire to be exposed to VR scenarios in the future and would recommend the experience to their loved ones and friends.

Overall, seven main themes were identified based on what participants verbally shared during the experience (see Table 4): (1) reminiscence (11/22 participants; 47.8%); (2) aesthetic appreciation of the virtual context (8/22 participants; 34.7%); (3) realism (6/22 participants; 26.1%); (4) sense of safeness and protection in the virtual environment (4/22 participants; 17.4%); (5) suggestions for improving the VR scenarios (2/22 participants; 8.4%); (6) lack of interest (2/22 participants; 8.4%); (7) appreciation of the new experience (1/22 participants; 4.3%).

**Table 4:** Themes and quotes by participants.

Main theme	User quotes
“Reminiscence”	“(…) there is also my mom, she is old now (…) we went together to the see (…)” (Participant 2)
	“I was with my dad at the sea—I went to the sea” (Participant 2)
	“ (…) my mountain (…)” (Participant 3)
	“I walked a lot in my mountains” (Participant 4)
	“We went there with the children” (Participant 6)
	“We used to play on a field like this” (Participant 6)
	“When I could walk in the mountains (…) melancholy” (Participant 5) (then she cocked up by remembering what she is no longer able to do)
	“I went to the sea in Jesolo, good memories. Now the children go alone” (Participant 9)
	“(…) in Sardinia, this sea reminds me of my happy time” (Participant 10)
	“I took care of my garden” (Participant 20)
	“I went many times in Sicily with children” (Participant 15)
	“How I liked to walk in the mountains” (Participant 14)
	“ (…) I remember lots of things (…)” (Participant 22)
“Aesthetic appreciation of the virtual context”	“We went to the mountains with blankets (…) and everyone sang the mountain songs” (Participant 16)
	“ (…) beautiful here” (Participant 3)
	“Suddenly a beautiful snowfall (…) the trees are beautiful (…) there are little animals and butterflies that go around (…) is beautiful (…) beautiful trees and the river that moves split in two—the effect is more positive than negative” (Participant 4)
	“Beautiful” (Participant 12)
	“Beautiful place” (Participant 13)
	“Wow(…) how beautiful (…) what beautiful streams” (Participant 14)
	“​​Beautiful, beautiful (…) that wonder” (Participant 15)
	“Beautiful, the sea is immense, what a wonder, I see clouds” (Participant 22)
	“Beautiful that stuff (…) how did you do, beautiful, but is there such a place?” (Participant 22)
“Realism”	“There’s a wad there that’s gone because I touched it” (Participant 7)
	“Touch the water of the sea (laughs) (..) oh maria looks at the beautiful trees (..) just bathe the water” (Participant 10)
	“I can't see well what it looks like, it seems cloudy but it is clear they are not” (Participant 18)
	“They seem like the *Cascata delle Marmore*” (Participant 15)
	“Does not increase the wind that takes me away” (Participant 15)
	“(…) seem like scarecrows that make blue flowers” (Participant 15)
	“Now comes the water, (…) it makes me feel good—but is it true?—look how much water that comes” (Participant 16)
	“Some things don’t seem true (…) plant shadows don’t move (…)” (Participant 13)
“Sense of safeness and protection in the virtual environment”	“I feel calm here” (Participant 2)
	“I feel good” (Participant 5)
	“I feel safe” (Participant 8)
	“The sea is the place that makes me feel good and safe even in reality” (Participant 16)
“Lack of interest “	“I’d rather go back to my room (…) I saw that birds pass by and that’s it, I like all the tools (annoyed), I’m fine everything it’s nice but nothing more, I want to go back to my room” (Participant 1)
	“I don’t care so much today, I’m old, a little heavy (referred to the HMD)” (Participant 19)
“Suggestions”	“Beautiful, but I would like more people here” (Participant 7)
	“Plants must be better arranged” (Participant 9)
“Appreciation of the new experience”	“Nice to see new things” (Participant 8)

### 2^ objective: Preliminary investigation of the customized VR scenarios on emotional states investigated before, during, and after the virtual exposure

Based on the modified version of the STAI-Y1, a Wilcoxon Signed Ranks Test revealed that scores related to a state of relaxation were significantly higher after the VR experience (Md = 3, *n* = 22) than before (Md = 2, *n* = 22), z = 2.29, *p*-value = 0.022. Moreover, levels of manifested anxiety and worry significantly decreased after the experience (Md = 1, *n* = 22) if compared to levels found before the virtual exposure (Md = 2, *n* = 22), z = -3.13, *p*-value = 0.002. No differences were found for sadness, tightness, annoyance, and anger (− 1.73 < z < − 0.88; *p*-value > 0.05). (see Table 5).

**Table 5:** State emotions comparisons.

	T0 (before VR)			T1 (after VR)		
	25th percentile	50th percentile (Median)	75th percentile	25th percentile	50th percentile (Median)	75th percentile
Relaxed	2	2	3	2	3	4
Sad	1	1	1.25	1	1	2
Uptight	1	2	3	1	1	2.25
Annoyed or upset	1	1	2	1	1	1.25
Worried/Anxiety state	1	2	3	1	1	2

**Figure 4:**
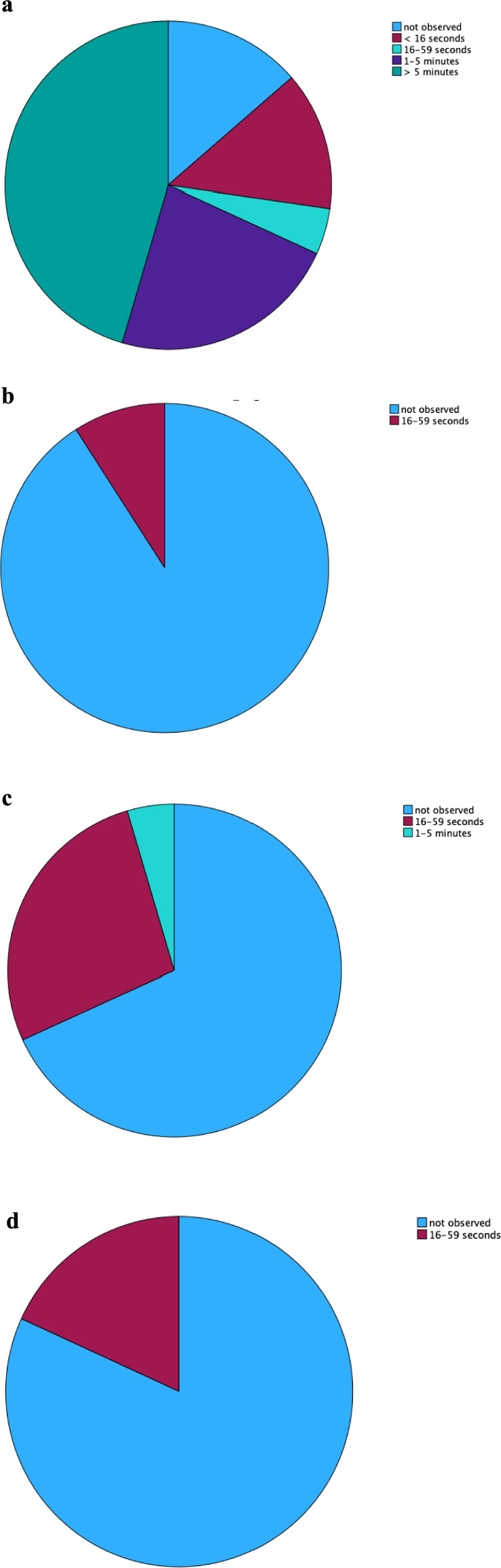
**(a, b, c, d).** Pie charts related to the frequency of emotional states recorded with the OERS. (**a**) Pie chart related to the frequency of “enjoy” recorded with the OERS. (**b**) Pie chart related to the frequency of “anger” recorded with the OERS. (**c**) Pie chart related to the frequency of “worry-fear” recorded with the OERS. (**d**) Pie chart related to the frequency of “sadness” recorded with the OERS.

More than half of participants (12/22; 52.2%) expressed being engaged and involved in the virtual environment. During the VR experience, it was observed that 10/22 (43.5%) participants felt a state of pleasantness for more than 5 min, 5/22 (21.7%) seemed to enjoy the activity for a period between 1 and 5 min, 1/22 (4.3%) for at least a minute, 3/22 (13%) less than 16 s and a minority, 3/22 (13%), reported not enjoyable manifestations (see Fig. 4).

Only two participants (8.7%) showed a state of annoyance for at least a minute, Six users (26.1%) showed to be worried during the virtual activity for at least a minute, and 1/22 (4.3%) for at least two minutes. Four participants (17.4%) expressed sadness for no more than a minute (see Fig. 4).

### 3^ objective: Preliminary investigation of the usability of the VR procedure and apparatus as perceived by the health staff

Seven individuals from the health staff provided assistance during the VR sessions (at least two VR sessions each). At the end of the entire VR experimental procedure with participants, 5/7 health staff operators took part in a dedicated assessment phase focused on assessing the VR procedure's usability from their individual perspectives and in a focus group.

Based on the SUS questionnaire, all participating staff rated the technology is equal to or above 67.5 with an average score of 69 (Standard Deviation = 2.24; min. = 67.5; max. = 72.5), which corresponds to a level of “marginal” acceptance and usability, defined as “OK” ^47,48^. The score is associated with the fact that health staff operators, based on the observation of the procedure performed by the experimenter with the support of the technologist, would only use it less frequently as they consider the system complex to use independently without the presence of a technician. Moreover, the score reflects their lack of confidence in administering the procedure alone. In general, the low SUS score regards the technical difficulties expected by operators in administering it independently. The main difficulty was the use of the PC dashboard for setting up the environment. For this reason, the following study that we will carry out in which operators will administer the procedure directly to patients will involve the use of an easy to use smartphone interface for customizing the VR environments without the support of a technician.

The health staff participants stressed that the support of a technical person would be needed to ensure the successful implementation of the VR-scenarios, especially due to the role the PC plays in customizing the environments.

The A-MARS—Section E scale was used to assess the subjective quality rating of the VR apparatus. All the health staff individuals reported that they would recommend the VR apparatus to several people that could obtain benefit from its use. Most of the staff respondents (3/5) were willing to use the VR solution at least 10–50 times in a year, and participants were willing to pay for such a product. When requested to evaluate the overall experience they had with the VR solution, healthcare staff participants expressed an average score of 3.5 points on a scale from 1 ("*One of the worst e-tools I've used*") to 5 ("*One of the best e-tools I've used*"). Finally, all healthcare staff said that they thought the use of VR was useful in facilitating relaxation, especially in patients with mild cognitive impairment, since people with more severe impairment may find additional obstacles in experiencing VR (e.g., related to the difficulty in understanding what objects represented).

The focus group was aimed at investigating five main themes related to the VR apparatus usage. For each theme, at least two questions were asked, and the following themes were derived:

- Theme 1: **Strengths and advantages of VR use**:

(1.1: "*According to your experience, are there strengths and advantages in using virtual reality as a pleasant activity to promote relaxation in people with cognitive impairment?";* 1.2: *"What are they all about?*").

All the participants agreed in judging the VR apparatus and procedure as a simple activity to do with users since it does not require particular cognitive or motor skills. Health staff individuals thought it was a valuable activity for promoting pleasant and potentially relaxing experiences in older people with various levels of cognitive impairment. Another advantage they saw regards the VR capacity to allow hospitalized people to see places they can no longer experience. Another strength from the health staff's point of view is the possibility of exploring a different kind of virtual context and choosing the preferred scenarios according to the users’ preferences and needs.

- Theme 2: **Weaknesses and disadvantages of using VR**:

(2.1: "*Based on your experience, are there disadvantages and weaknesses in using virtual reality as a pleasant activity for promoting relaxation in people with cognitive impairment?";* 2.2: *"What are they all about?*").

All participants agreed that the heaviness and wearability of the Oculus quest-2 HMD, or the need to wear it, was the main obstacle in the VR procedure and the primary reason why some people refused to participate in the activity. According to 3/5 health staff professionals, the structured device was seen as unsafe, especially for people with higher cognitive impairment or concerned about their safety.

- Theme 3: **Future perspectives and what to change for continuing the use of VR with the health facility's users**:

(3.1: *"Based on your experience, should the virtual reality procedure be modified to continue to be used with people with cognitive impairment?";* 3.2: *"How?*").

All health staff individuals agreed in suggesting to change the type of HMD, going for models that are lighter and more wearable. For people with higher cognitive impairment, it would be difficult to understand the reason for wearing the HMD, and this may also elicit some anxiety. A less invasive HMD may likely help to overcome these difficulties. A suggestion was to consider exposing users that perceive higher anxiety levels in wearing the HMD, and greater difficulty understanding the activity, in a virtual setup that does not require to wear the HMD (e.g., a Cave Automatic Virtual Environment).

All participants agreed to actively involve the health staff in the VR deployment with users to make the context even more familiar.

Another element to consider for the future is to expand the number of virtual reality experiences to familiarize the person with the equipment and the virtual context.

Three health staff suggested creating other VR environments and making natural contexts more realistic. Another suggestion for future investigation was to deploy the procedure without using the PC.

- Theme 4: **Facilitating factors of the VR procedure deployment**:

(4.1: "*Based on your experience, are there any factors that have facilitated the use of virtual reality?;* 4.2: *"What are they all about?*").

All participants said that a facilitating factor could be the deployment of the VR procedure in a familiar setting to facilitate users' perception of safety.

One health staff member added that, regardless of the severity of the cognitive impairment, having peculiar personality characteristics (e.g., being predisposed to new experiences) could be another facilitating factor. From a general perspective, health staff participants agreed in considering low levels of cognitive decline as a condition that might decrease the benefit provided by the VR intervention (e.g., for issues related to understanding the intervention’s aim, and for the difficulty in expressing and understanding if the activity is really appreciated). However, they reported that the most important human factor to consider when inviting to a VR session is related to the individual personality characteristics, since they observed how two participants—despite a critical level of cognitive impairment—were calm and positive during the VR exposure.

- Theme 5: **Risks in using virtual reality**:

(5.1: "*Based on your experience, are there risks in applying this technology the way it was done in this research?";* 5.2: *"What are they all about?*").

The most significant risk encountered is eliciting intense emotions, not only positive, that must be contained even after exposure to virtual reality. They also considered this a potential point of strength of the solution since it allows people to be exposed to their feelings and memories. Based on the experience gained during the VR sessions, they did not believe there were any further risks as the procedure was assisted.

From the focus group transcript, information on the frequency of agreement and disagreement expressed directly or through examples from each participant was collected for questions 1.1, 2.1, 3.1, 4.1, and 5.1 and was systematically described in Table 6 following the example of previous studies (e.g., ^40,51^).

**Table 6:** Frequencies related to the agreement/disagreement expressed by the health care staff participants to the main themes of the focus group.

Question	Type of response					
	A	SE	NR	D	SD	AR
1.1 *Are there strengths and advantages in using VR as a pleasant activity for promoting relaxation in people with cognitive impairment?*	4	1	0	0	0	0
2.1 *Are there disadvantages and weaknesses in using VR as a pleasant activity for promoting relaxation in people with cognitive impairment?*	5	5	0	0	0	0
3.1 *Can the VR procedure be modified to continue to be used with people with cognitive impairment?*	3	2	0	0	0	0
4.1 *Are there any factors that have facilitated the use of VR?*	1	3	1	0	0	0
5.1 *Are there risks in applying this technology the way it was done in this research?*	3	1	1	0	0	0

**A**: Explicit agreement; **SE**: Provided example suggesting agreement; **NR**: Did not indicate agreement or dissent (i.e., nonresponse or did not know);** D**: Indicated dissent; **SD**: Provided significant example suggesting dissent; **AR**: Ambivalent response.

## Discussion

The core objective of the present study was to assess the feasibility of and potential benefit of VR exposure in a target of older adults living in long-term care. From a general perspective, feasibility and acceptance proved to be satisfactory, in line with previous research (e.g.,^18,19^): the exposure to the VR apparatus was well-tolerated, and no severe or notable adverse side effects (such as nausea, dizziness, or confusion) were reported. The most frequently reported symptoms by only those who wore the VR apparatus for more than five minutes were a slight sensation of fatigue and eye discomfort limited to the period of exposure in the HMD. It is relevant to note that the VR environments used in this study are labeled as static scenarios with only slight visual oscillations (e.g., seawater and oscillation of plants due to the action of the wind), but without any rapid movement. This was an intentional design consideration, given that previous work reported that static scenes and a constant speed during movement could prevent cybersickness symptoms (e.g.,^52–55^).

The main difficulty referred to by nearly half of the participants (both patients and healthcare staff) was the weight and wearability of the HMD. This issue has been previously reported, for instance in Kalantari et al. study^17^ but differs slightly from older studies (e.g.,^18^) where the model of HMD used (Samsung Gear VR) only weighed 318 g, while Oculus quest 2, used here, weighs 503 g. Even if a larger sample is necessary to consider these results representative of the target population, our sample was entirely composed of older adults, individuals with a slender physique and weak muscle mass, which undoubtedly contributes to the perception of heaviness. Despite this limitation, the perceived weight of the device by the participants did not significantly affect the overall experience of the virtual context or withdrawal from the study.

Similar to other studies (e.g.,^17–19,56^), participants expressed verbally or manifested with nonverbal behavior a general state of interest, awareness, engagement, and enjoyment during the VR exposure showing appreciation of the experience. Reports collected during the intervention showed that the exposure to a realistic, natural, and above all, customized virtual environment adapted to users’ preferences and needs, prompted reactions, such as reminiscence of melancholic memories, the aesthetic appreciation of what they were seeing, the sense of safeness and protection in an appreciated context. Interestingly given the research context, participants also expressed feelings of self-realization and “being useful” by providing opinions and suggestions to the experimenters on how to improve the virtual scenarios.

Moreover, more than 70% of the participants stated that they would recommend VR to a friend, confirming the potential benefits of the VR experience in users with cognitive impairment.

The general increased relaxation state and the reduction in worry/anxiety post-VR, both self-reported and observed, are consistent with prior findings (e.g.,^18,19,57^), supporting the hypothesis that VR can serve as a restorative or stress-reducing experience.

A set of core constructs emerged from the focus groups conducted with healthcare professionals. In line with data reported by older adult participants involved in the study, one healthcare staff perceived the HMD to be too difficult and heavy for older adults. Even if corrective measures could be taken to improve comfort when wearing the HMD, novel and lightweight HMD might further facilitate the acceptance and ease of use, especially for those in a more fragile state.

A second constraint related to the difficulty of properly assessing a patient's VR experience in case of severe cognitive impairment. In this case, the collection of bio-physiological parameters could be added as part of the experimental procedure, to better assess the experience of the patient and triangulate different sources of information (e.g., heart rate, skin conductance, etc.). Finally, the collection of informed consent was reported as a problematic—albeit necessary—task: signing (pen-and-paper) a form was per se not well accepted by older adults, whilst other approaches of collecting informed consent might be more suitable and easy-to-deploy (e.g., verbal consent). It is, however, important to add that this step is essential for the deployment of a research project, but it is expected it would not impact the implementation of a therapy program.

Considering the major (perceived) advantages of using VR for patients, focus group participants highlighted the possibilities to deploy environments patients are not able to experience anymore (e.g., seascapes, mountainscapes), because of clinical and/or logistical issues.

Given the feedback provided by staff during the focus group, and the results from patient participants using VR, we provide several recommendations to inform future developments and improvements of the solutions, both from a methodological viewpoint and an operational perspective.

From an operational perspective, the HMD proved to be generally acceptable but a lighter-weight HMD might improve usability, particularly for this target group. In addition, to mitigate discomfort and potential anxiety, identifying customized time and space could be beneficial to meet patients’ needs. For instance, certain times of day (e.g., sundown syndrome) and locations (unfamiliar rooms) may trigger anxiety and behaviours, which should be avoided when necessary. Finally, preparatory procedures should include a targeted and careful assessment of physical condition, including hearing aids, glasses, and mobility devices, although, in the current research, no major problems were reported.

From a methodological perspective, enriching the VR experience with additional audio and video details could further improve the impact of the VR intervention, considering that patients might be more familiar with some environments (e.g., mountain scenery) over others, and thus paying more attention and wanting to explore specific details.

In terms of customization, this option appeared to be appropriate and viable for the target group. In fact, the core objective was to explore the feasibility of the entire procedure (including the availability of certain degrees of customization), as this option could potentially be neither viable nor acceptable for this context.

Adding psycho-physiological measures (e.g., heart rate variability, skin conductance, etc.) could greatly improve the assessment of anxiety/relaxation, particularly in patients with severe cognitive impairment, where there may be challenges in communicating. It is important to acknowledge that participant anxiety levels in this study may have been higher than expected because patients were exposed to VR for the first time, and in an unfamiliar room (which was dedicated to this study). An additional methodological issue is related to participants' sample inclusion criteria. In the large majority of studies, measures that evaluate the cognitive decline level (e.g., the MMSE) are used as reference (e.g.,^18^). In our study we demonstrate that individuals of all ranges of cognition are able to benefit from VR. Even if we did not investigate personality traits based on proper measured, qualitative consideration reported by the observers during the Focus Group suggests that personality traits might play a role in reinforcing efficacy of these interventions. Despite the fact that they were not specifically measured in this study, authors might argue that some specific traits (like willingness to try new experiences) could be a reliable proxy of potential success, therefore these characteristics should be considered (perhaps weighted even more) than cognitive abilities in future studies to properly assess their impact and role in the process.

Another important outcome of this study highlighting the potential of VR customization. In terms of acceptance, personal-adaptation refers to the ability to tailor environments and features to users’ preferences (e.g., removing personal triggers for anxiety related to peculiar visual and auditory stimuli that could characterize the VR scenarios). For efficacy, adaptation implies the opportunity to facilitate stronger connection between the virtual context and a personal history or narrative. In other words, linking the virtual environment with personal constructs/meanings could facilitate the emergence of viable and sustainable “therapeutic” experiences, where the patient is involved somewhere in between the area of elaboration, even considering the reminiscence, that can emerge during the exposure and may contribute to decreasing anxiety^58^.

### Limitations

While promising, it is important to underline several potential limitations that may have introduced bias in the present study. Given the small sample size and recruitment from a single long-term care facility, generalizability of the results should be taken with caution. Moreover, even if a recent study did not evidence gender differences in VR’s user experience, presence, and usability^59^ gender-related differences are still debated in the literature^60,61^. For this reason future studies should stratify the samples to control the effect of various socio-demographic variables, such as gender. Additional bias could be introduced through subjective researchers' observations, and the lack of triangulation of self-reports with more objective measures, such as biophysiological data. Authors cannot exclude that an increased level of interaction (e.g., combining metaverse and game mechanics)—not included in this study—could have an impact on the potential acceptance of the proposed experience. In addition, a limitation could be represented by the lack of neural networks to further analyze the different sources of data: this would be possible only with large datasets, and this limitation is directly linked to the purpose of the study and the related sample size.

An additional limitation is linked to the relatively limited range of customization options made available: they were limited as this range was deemed appropriate for the target group of the study, but could be appropriate to suggest more focused stimuli very close to the personal life, interest, values and needs of each user. Finally, because we used an adapted version of the STAY questionnaire (to suit our population of interest^18,19^), we cannot ensure the validity of the tool.

### Future directions

Future research should compare different HMDs (e.g., PICO 4 HMD^62^) and weights (among other features—more easily applied to vision glasses etc.) to see if this has a noticeable impact on tolerability by older adults. VR-content should also be enriched with added audio and video details. Future studies could focus on widening the range of customization options in the virtual scenarios and on measuring their impact, extending the contribution and knowledge provided by our study.

Studies should assess the impact of multiple exposures, both in terms of acceptance and the clinical effect on anxiety. With this approach, healthcare professionals could administer VR several times to the same individuals, which may decrease feelings of fear, anxiety, and prejudice related to the unfamiliar technology^57^. It also allows researchers to evaluate the potential impact of the intervention at different moments of the day^56^.

Considering the emotional impact VR sessions could evoke, future intervention should design pre and post-strategies to help mitigate or manage cognitive/emotional reactions potentially triggered by the VR. For example, the introduction of listening spaces, dedicated space to contain emotional states. A more focused analysis about the specific diseases would be useful to better understand if, for example, some but not other visual and auditory problems prevent ease-of-use of the HMD. Stratifying the sample according to the severity of cognitive deterioration to better assess if the VR apparatus deployed could be more effective for some groups over others, as well as to investigate the possible changes to the virtual context in relation to users’ preferences and needs. Finally, in terms of increasing the evidence-base, studies should recruit a larger sample size and include objective psycho-physiological assessments. An increased sample could also provide the opportunity for applying more sophisticated data analysis techniques to assess the impact of this technology and stratify the subjects for different characteristics.

## Conclusions

This study builds on the growing body of literature empirically evaluating the acceptance and potential impact of immersive VR in long-term care settings, with specific considerations for older adults with varying degrees of cognitive, sensory and mobility impairments. Based on our knowledge, unique to other work, we took into account the effect of customizing the VR-experience. This represents a valuable step-forward compared to the standard “one-size-fits-all” approach adopted in the majority of the studies in the field of VR^63^. At the same time, while customization could improve acceptance by accommodating and tailoring the VR environment in light of patients’ preferences (e.g.,^30,31,33^), it might lead to potential challenges in its deployment. This factor could play a key role in either facilitating or hampering the experience and to our knowledge this is among the very few studies exploring customization as an option for this kind of population. Since user-centred approaches are key for developing more effective solutions, it is also critical that we seek the input of stakeholders, such as those responsible for providing therapeutic support, in the design of novel forms of intervention to ensure their sustainable adoption (e.g.,^63^).

In this study, we did consider the viewpoints of healthcare staff which brought valuable insight into the patient's previous history and usual emotional patterns and reactions. Moreover, we were able to correlate feedback between patient and staff groups, and across the multiple data collection methods.

Despite few limitations, this research contributes to advancing knowledge related to the customization of VR experiences and their related impact on acceptance and potential clinical efficacy in older adults. Given the recognition that current interventions are not satisfactory and that the number of older adults with cognitive decline will only increase, we need to dedicate resources to the co-design and evaluation of novel therapies for this population. VR presents a relatively cost-effective and customizable solution that can be deployed in long term care contexts, offering new hope for personalized-care (Supplementary Information).

### Supplementary Information

Supplementary Information.
