# Supplementary material for: Customized virtual reality naturalistic scenarios promoting engagement and relaxation in patients with cognitive impairment: a proof-of-concept mixed-methods study
Source: Sci Rep. 2023 Nov 22;13:20516. doi: 10.1038/s41598-023-47876-1 (PMC10665464; doi:10.1038/s41598-023-47876-1)
Supplement: Supplementary file 1 — Supplementary Information. [file 41598_2023_47876_MOESM1_ESM.docx]

**Project summary**

**Personalized Virtual Reality Naturalistic Scenarios Promoting Engagement and Relaxation in Patients With Cognitive Impairment: a Proof-of-concept Mixed-methods Study**

**Rationale & background information**

The impact of customizing VR scenarios is growing, showing different kinds of positive effects, such as an increased sense of presence and engagement in the virtual environment. A relaxing and customizable VR environment could allow the management of any interfering environmental factors that might also be present in the natural context. Additional investigations are still needed to obtain more consistent data on its feasibility and effectiveness.

Some published research has highlighted how using VR HMD promotes the management of depressive and anxious symptoms in older people. Appel et al. (2020) investigated the relationship between relaxing, positive environments and both positive and negative emotions based on a sample of older adults with varying (mild to severe) cognitive and physical impairment levels. The participants were exposed through an HMD for an average variable period of 8 minutes to a relaxing environment in which realistic natural scenarios were depicted. All 66 participants completed the research without experiencing or reporting adverse side effects related to using the HMD. Qualitatively, positive feedback is associated with the experience and the perception of a higher state of relaxation. Additionally, there has been an increase in the intensity and frequency of positive emotions experienced (e.g., feelings of relaxation and happiness) and a reduction in the levels of some negative emotions (e.g., sadness and anxiety). The authors concluded that exposure of people with cognitive and physical impairments to realistic and natural immersive scenarios in virtual reality through an HMD is safe and feasible. These findings encourage future studies to investigate the role of virtual reality scenarios’ personalization.

**Study goals and objectives**

Considering this evidence, the current proof-of-concept study is based on the following goals:

evaluate the impact of VR on self-reported and observational levels of motion-sickness, engagement, and pleasantness in older adults living with cognitive impairment and residing in long-term care.

investigate if personalized, relaxing virtual environments can positively impact feelings and state anxiety.

investigate the VR apparatus's usability from the health staff's perspective.

The goal of this feasibility study is to investigate the feasibility of a personalized naturalistic Virtual Reality scenario by assessing motion-sickness effects, engagement, pleasantness, and emotions felt considering a sample of individuals with cognitive impairment resident at the Azienda Pubblica di Servizi alla Persona (APSP) "Margherita Grazioli", a long-term care home in Trento (Italy) in collaboration with the Department of General Psychology - University of Padova (Italy) and the Centre for Health and Wellbeing-Fondazione Bruno Kessler (Italy). The current proof-of-concept and feasibility study is a one-session single-centre trial based on a mixed-methods approach inspired by the Obesity-Related Behavioral Intervention Trials (ORBIT) framework for the design (Phase Ib) of digital interventions and their preliminary testing (Phase IIa).

**Study Design and Methodology**

**Arms and Interventions**

Personalized Virtual Reality exposure: Participants are exposed to a personalized, relaxing VR scenario administered by the Oculus Quest 2 tool.

The administration was deployed in one session.

**Outcome Measures**

Primary Outcome Measure:

1.Assessing the general and eye-related physical symptoms of exposure to a virtual reality environment.

To assess these symptoms, the Virtual Reality Symptom Questionnaire (VRSQ) was used. The score assigned to each item ranges from 0 to 6, with a maximum total score of 84 (48 for general symptoms and 36 for eye symptoms). Higher scores represent worse symptoms, with 0 corresponding to no adverse effects, and 84 to serious adverse effects.

[Time Frame: Through study completion, an average of 6 months]

2.VR experience tolerability estimated based on the frequency of time spent in the VR context.

The VR experience tolerability was estimated based on the frequency of time spent in the VR context.

[Time Frame: Through study completion, an average of 6 months]

3.Usability of the VR apparatus

The usability has been investigated by a measure developed and described by Appel et al. (2020) composed of both self-reported questions and other queries that the experimenter answered by observing the participant's behavior during the experience and characterized by a series of items based on a 5 points Likert scale (Questions about Level of interest, awareness, engagement, and enjoyment observed: 1="very much", 5="not at all"; min. score=5 and max. score=25; higher scores mean a worse outcome. Questions on other information in relation to the VR experience: 1= "strongly disagree", 5= "strongly agree"; min. score=17 and max. score=85; higher scores mean a better outcome), and six open-ended questions focalized in obtaining additional information, where possible, about: 1) what participants liked best and least; 2) what participants would like to see; 3) if participants would like to repeat the experience; 4) if participants would recommend the experience to a friend.

[Time Frame: Through study completion, an average of 6 months]

Secondary Outcome Measures:

Change from before and after the one-shot VR session in the relaxation using the modified version of the State-Trait Anxiety Inventory-Y1 (STAI-Y1).

The modified version of the State-Trait Anxiety Inventory-Y1 (STAI-Y1), inspired by Appel et al.16,17, was administered in this study to obtain information on the state-anxiety level experienced before and after the VR session.

The scale was administered as an interview based on a 5-point Likert scale from 1 (not at all) to 5 (a lot); min. score=15; max. score=75. Higher scores mean a worse outcome.

[Time Frame: Through study completion, an average of 6 months]

Change from before and after the one-shot VR session in emotions felt using the Observed Emotion Rating Scale (OERS).

The Observed Emotion Rating Scale (OERS) was adapted from the original version of Lawton et al.41 and used as an observation tool to assess the presence and frequency of negative emotions (fear, anxiety, anger, and sadness) and positive (pleasure) feelings experienced during the session based on a scale from 1 to 5 (1: "undetected emotion"; 2: "emotion observed for less than 16 seconds"; 3: "emotion observed for 16-59 seconds"; 4: "emotion observed for 1-5 minutes"; 5: "emotion observed for more than 5 minutes").

[Time Frame: Through study completion, an average of 6 months]

Feedback about the perceived quality rating of the VR set-up deployed from health care staff

To obtain information from health professionals about the perceived quality rating of the VR set-up deployed, the Adapting-Mobile App Rating Scale (A-MARS)-Subjective Quality Scale was filled in by operators who participated during the VR sessions. The administration was performed at the end of the administration of the experimental procedure. (Likert scale from 1 to 5; min.score=4 and max.score=25; higher scores mean a better outcome).

[Time Frame: Through study completion, an average of 6 months]

Feedback about usability from health care staff

To obtain information from health professionals about usability of the VR set-up deployed, the System Usability Scale (SUS) was filled in by operators who participated during the VR sessions. The administration was performed after the administration of the experimental procedure. The SUS consists of a 10 item questionnaire on a 5-points Likert scale (1: Strongly agree; 5: Strongly disagree). The score for each question is converted to a new number, added together and then multiplied by 2.5 to convert the original scores of 0-40 to 0-100. Higher scores mean a better outcome.

[Time Frame: Through study completion, an average of 6 months]

Feedback about acceptability from health care staff

To obtain information from health professionals about acceptability, at the end of the experimental phase, a focus group was conducted with the health professionals that assisted users during the VR experience. Issues discussed during the focus group were the strengths and weaknesses associated with using virtual reality, the future perspectives, and the risks associated with using virtual reality with users affected by cognitive impairment.

[Time Frame: Through study completion, an average of 6 months].

**Eligibility**

Minimum Age: 18 Years

Maximum Age: 100 Years

Sex: All

Gender Based: No

Accepts Healthy Volunteers: No

Criteria:

Inclusion Criteria:

Italian mother tongue

Clinical diagnosis of Cognitive Impairment (mild, moderate, or severe).

Exclusion Criteria:

Palliative care

Clinical diagnosis of psychosis

Severe neurological damage

A clinical diagnosis of epilepsy (or having first-degree relatives diagnosed with epilepsy) - - Cardiac pacemaker or other metal devices

Infectious or gastrointestinal disorders

Open wounds at the level of the face

Motor or visual dysfunctions and neuromuscular pain that prevent the use of Oculus.

**General information**

- Protocol title: Personalized Virtual Reality Naturalistic Scenarios Promoting Engagement and Relaxation in Patients With Cognitive Impairment: a Proof-of-concept Mixed-methods Study; protocol identifying number: NCT05863065 (08/05/2023).
- Sponsor: University of Padova
- Collaborators: Fondazione Bruno Kessler, Azienda Provinciale per i Servizi Sanitari, Provincia Autonoma di Trento, TrentinoSalute4.0
- Principal Investigators: Caterina Novara (caterina.novara@unipd.it), Susanna Pardini ([susanna.pardini@phd.unipd.it](mailto:susanna.pardini@phd.unipd.it)) - University of Padova.
- Central Contact Person: Susanna Pardini, PsyD; Telephone: +393335944315 Email: susanna.pardini@phd.unipd.it
- Locations: Azienda Pubblica di Servizi alla Persona (APSP) "Margherita Grazioli” Trento, TN, Italy. Contact: Patty Rigatti 0461/810688 [p.rigatti@apspgrazioli.it](mailto:p.rigatti@apspgrazioli.it). Contact: Susanna Pardini 3335944315 [susanna.pardini@phd.unipd.it](mailto:susanna.pardini@phd.unipd.it), University of Padova, Padova, PD, Italy, 35134. Contact: Susanna Pardini, PsyD 3335944315 susanna.pardini@phd.unipd.it

### Safety and ethical considerations

### From a scientific point of view, all the data, parameters, and management methods considered in this study will be identified by a scientific-technical committee composed of health professionals/contact persons of the centers involved in the study. Finally, participants who decide not to participate in or leave the study will not see any change in the quality of care by the physician or the service to which they belong, according to the standards currently implemented in APSP “Margherita Grazioli”. To safeguard patients and given the exploratory purpose of this research, the exclusion criteria applied cover a wide range of clinical and contextual characteristics for the patient, even if only potentially risky in case of exposure to virtual reality (palliative care, diagnosis of psychosis, severe neurological damage, a positive diagnosis of epilepsy or having first-degree relatives diagnosed with epilepsy, cardiac pacemakers or other metallic devices, infectious or gastrointestinal disorders, presence of open wounds in the face, motor or visual dysfunctions and neuromuscular pain that prevent the use of Oculus). In any case, it will be expected a moment of training and accustoming to virtual reality before the actual exposure of about 1-2 minutes.

### Data management and statistical analysis

The experimental processing will be carried out with the help of paper and automated processes, to ensure the data's confidentiality, integrity, and availability. Signed consent forms and the study log (patching participant names to study ID) will be processed and stored based on a pseudo-anonymized form exclusively for the aforementioned purposes by the authorized personnel of the Department of General Psychology - University of Padua and APSP “Margherita Grazioli”.

All observational notes will be collected anonymously. All data collected during the VR exposures will only be linked to the participant’s study ID. Biomarkers during the VR exposures will be transmitted directly from the headset sensors to a secure SD card that will be stored in Dr. Christopher Smith’s office.Sample size estimation.

Sample size calculations were performed based on proof-of-concept study protocols: expected medium standardized effect size (effect size = .65), with a power of 80%. Sample size and Power for non-parametric tests (Kruskal-Wallis Test, Wilcoxon test) were calculated with G-Power. Results suggest that, with non-normal distribution, 22 patients have to be enrolled. If parametric statistics are conducted (assuming the normal distribution), the sample should instead consist of n = 21 users with Bonferroni correction (alpha = 0.05), an effect size equal to .65, and a power of 80%.

Data analysis.

Continuous data will be presented as mean ± standard deviation. Categorical data will be presented as numbers with percentages. The statements made by participants during the VR experience will be analyzed using thematic analysis and reported as frequencies. Data will be analyzed thematically following an inductive, data-driven approach. Data codes will generated systematically, collated into themes, and applied to the data set to generate frequencies. For a comparison of non-normally distributed continuous variables before and after the VR intervention, the Wilcoxon signed-rank test will be used for each intervention separately. A two-sided p-value <.05 is considered significant. Statistical analyses were undertaken using SPSS® version 29. Moreover, qualitative responses will be transcribed before, during, and after the VR exposure. In order to analyze data obtained by the audio-recorded focus group, the micro interlocutor analysis method will be applied. The present method is helpful in obtaining information on participants’ attitudes, points of view on the use of VR, and permits to have quantitative data on participant grouping. Data were analyzed based on descriptive statistics.

### Duration of the project

### Study Start: January 23, 2023

### Primary Completion: March 6, 2023

### Study Completion: March 6, 2023

**References**

[1] Lim, P.Y., Dillon, D. & Chew, P.K.H. A Guide to Nature Immersion: Psychological and Physiological Benefits. *Int. J. Environ. Res. Public Health* **17**(16), 5989. doi: 10.3390/ijerph17165989 (2020).

[2] Park, S.H. & Mattson, R.H. Ornamental indoor plants in hospital rooms enhanced health outcomes of patients recovering from surgery. *J Altern. Complement. Med.* **15**(9), 975-80. doi: 10.1089/acm.2009.0075 (2009).

[3] Jo, H., Song, C. & Miyazaki, Y. Physiological Benefits of Viewing Nature: A Systematic Review of Indoor Experiments. *Int J Environ Res Public Health.* **16**(23), 4739. doi: 10.3390/ijerph16234739 (2019).

[4] Ulrich, R. S. et al. Stress recovery during exposure to natural and urban environments. *Journal of Environmental Psychology* **11**(3), 201–230. [https://doi.org/10.1016/S0272-4944(05)80184-7](https://psycnet.apa.org/doi/10.1016/S0272-4944(05)80184-7) (1991).

[5] Benjamin, K., Edwards, N., Ploeg, J. & Legault, F. Barriers to physical activity and restorative care for residents in long-term care: a review of the literature. *J Aging Phys Act.* **22**, 154–65. doi: 10.1123/japa.2012-0139 (2014).

[6] Franco, L.S., Shanahan, D.F. & Fuller, R.A. A Review of the Benefits of Nature Experiences: More Than Meets the Eye. *Int J Environ Res Public Health.* **14**(8), 864. doi: 10.3390/ijerph14080864 (2017).

[7] Park, S.H. & Mattson, R.H. Ornamental indoor plants in hospital rooms enhanced health outcomes of patients recovering from surgery. *J Altern Complement Med.* **15**, 975–80. doi: 10.1089/acm.2009.0075 (2009).

[8] Park, B.J., Tsunetsugu, Y., Kasetani, T., Kagawa, T. & Miyazaki, Y. The physiological effects of Shinrin-yoku (taking in the forest atmosphere or forest bathing): evidence from field experiments in 24 forests across Japan. *Environ Health Prev Med.* **15**, 18–26. doi: 10.1007/s12199-009-0086-9 (2010).

[9] Lin, C.X., Lee, C., Lally, D. & Coughlin, J.F. Impact of Virtual Reality (VR) Experience on Older Adults’ Well-Being. In: Zhou, J., Salvendy, G. (eds) *Human Aspects of IT for the Aged Population. Applications in Health, Assistance, and Entertainment*. ITAP 2018. Lecture Notes in Computer Science, vol 10927. (Springer, Cham., 2018). <https://doi.org/10.1007/978-3-319-92037-5_8>.

[10] Riva G. Virtual Reality in Clinical Psychology. *Comprehensive Clinical Psychology* 91–105. doi: 10.1016/B978-0-12-818697-8.00006-6 (2022).

[11] Botella, C., Serrano, B., Baños, R.M. & Garcia-Palacios, A. Virtual reality exposure-based therapy for the treatment of post-traumatic stress disorder: a review of its efficacy, the adequacy of the treatment protocol, and its acceptability. *Neuropsychiatr. Dis. Treat.* **11**, 2533-45. doi: 10.2147/NDT.S89542 (2015).

[12] Maples-Keller, J.L., Bunnell, B.E., Kim, S.J. & Rothbaum, B.O. The Use of Virtual Reality Technology in the Treatment of Anxiety and Other Psychiatric Disorders. *Harv. Rev. Psychiatry* **25**(3), 103-113. doi: 10.1097/HRP.0000000000000138 (2017).

[13] Oing, T. & Prescott, J. Implementations of Virtual Reality for Anxiety-Related Disorders: Systematic Review. *JMIR Serious Games* **6**(4), e10965. doi: [10.2196/10965](https://doi.org/10.2196/10965) (2018).

[14] Carl, E. et al. Virtual reality exposure therapy for anxiety and related disorders: A meta-analysis of randomized controlled trials. *J. Anxiety Disord.* **61**, 27-36. doi: 10.1016/j.janxdis.2018.08.003 (2019).

[15] Kalantari, S. et al. Using a Nature-Based Virtual Reality Environment for Improving Mood States and Cognitive Engagement in Older Adults: A Mixed-Method Feasibility Study. *Innov. Aging* **6**(3), igac015. doi: 10.1093/geroni/igac015 (2022).

[16] Appel, L. et al. Older adults with cognitive and/or physical impairments can benefit from immersive virtual reality experiences: A feasibility study. *Frontiers in Medicine* **6**, 329. doi: 10.3389/FMED.2019.00329 (2020).
